# Autonomous lab-on-a-chip generic architecture for disposables with integrated actuation

**DOI:** 10.1038/s41598-019-55111-z

**Published:** 2019-12-30

**Authors:** Anke Suska, Daniel Filippini

**Affiliations:** Optical Devices Laboratory, Division of Sensor and Actuator Systems, IFM-Linköping University, S58183 Linköping, Sweden

**Keywords:** Lab-on-a-chip, Biomedical engineering, Applied physics

## Abstract

The integration of actuators within disposable lab-on-a-chip devices is a demanding goal that requires reliable mechanisms, systematic fabrication procedures and marginal costs compatible with single-use devices. In this work an affordable 3D printed prototype that offers a compact and modular configuration to integrate actuation in autonomous lab-on-a-chip devices is demonstrated. The proposed concept can handle multiple step preparation protocols, such as the enzyme-linked immunosorbent assay (ELISA) configuration, by integrating reagents, volume metering capabilities with performance comparable to pipettes (e.g. 2.68% error for 5 μL volume), arbitrary dilution ratio support, effective mixing and active control of the sample injection. The chosen architecture is a manifold served by multiple injectors ending in unidirectional valves, which exchange a null dead volume when idle, thus isolating reagents until they are used. Functionalization is modularly provided by a plug-in element, which together with the selection of reagents can easily repurpose the platform to diverse targets, and this work demonstrates the systematic fabrication of 6 injectors/device at a development cost of USD$ 0.55/device. The concept was tested with a commercial ELISA kit for tumor necrosis factor (TNF), a marker for infectious, inflammatory and autoimmune disorders, and its performance satisfactorily compared with the classical microplate implementation.

## Introduction

Autonomous lab on a chip (LOC) devices^1–6^ seek to extend the capabilities of LOCs beyond laboratories, thus diversifying their uses and reducing the limitations to operate and access such technology^7^. Different approaches aim at addressing this problem^2,3,8–11^, but for disposable microfluidic systems a central challenge remains for the integration of reliable actuation compatible with cost-effective manufacturing, as expected from a disposable element.

A key goal for any autonomous LOC concept is to support multiple step protocols, and a representative example of such situation is the classical enzyme linked immunosorbent assay (ELISA^12,13^), one of the most important formats of bioassays^14^. Accordingly, a platform capable of ELISA tests would cover a broad range of different assays, besides being able to directly reuse the abundant detection chemistries available for ELISAs.

**Figure 1:**
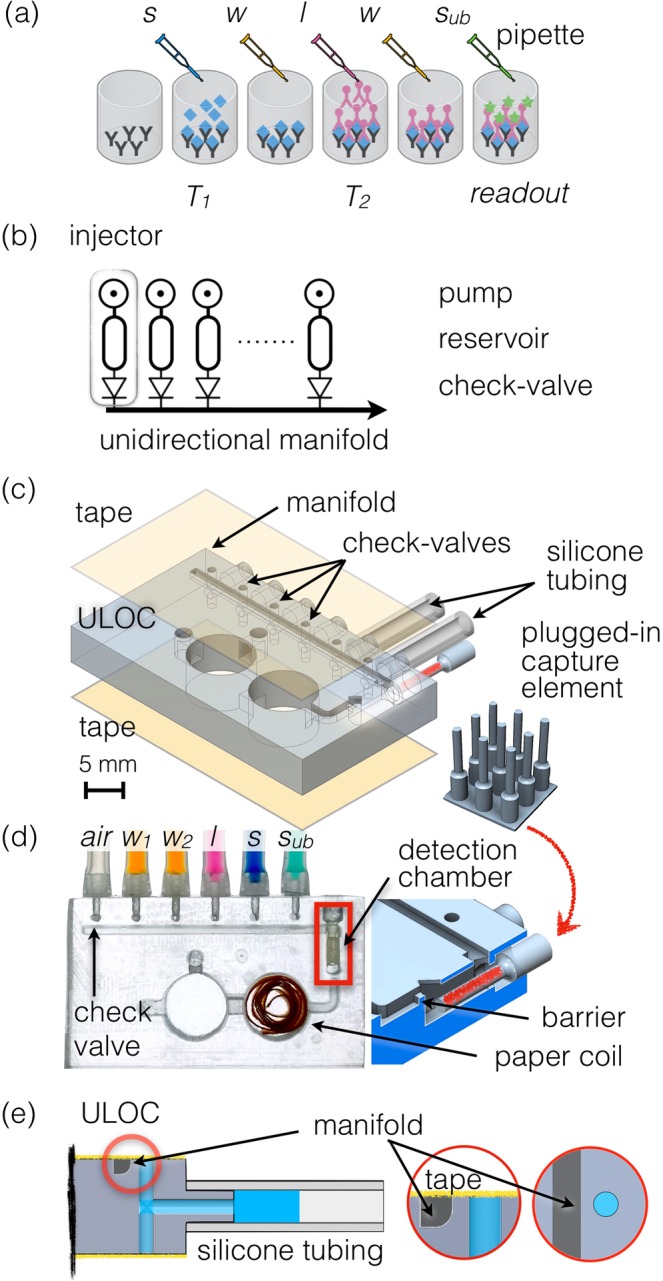
(**a**) Scheme of ELISA assay performed on a microplate. The sample (*s*) is pipetted into the functionalized well and after incubation (*T*_1_), washing buffer (*w*) is applied. Subsequently label (*l*) is pipetted and incubated (*T*_2_), followed by another washing procedure and a final delivery of enzyme substrate (*s*_*ub*_) for readout at a defined endpoint. (**b**) Scheme of the unidirectional manifold served by multiple injectors composed by a pumping element, a reservoir and a check-valve. (**c**) ULOC of 3D CAD highlighting the valve configuration and plugs to silicon tubing pumps, as well as the montage of the separately functionalized plugged-in capture element into the detection chamber, and the tape sealing. The inset show the separate 3D printout of the detection chamber elements. (**d**) Image of an assembled device ready for operation. The 3D model underscores the plug-in functional element and the geometric barrier assisting volume metering. (**e**) Detail of the injector with the check-valve body showing the 500 μm long barrier between the valve and the manifold, and the role of the sealing tape as elastic element to control unidirectional flow towards the manifold.

ELISA tests are dominantly performed on microtiter plates and evaluated with dedicated readers. The procedure demands trained operators, complementary tools and disposables for volume metering, a clean operation environment and a specialized readout system. In a typical ELISA test, sample and calibration concentrations are pipetted into functionalized wells (consuming one disposable tip in each operation), incubated for a given time; and after the removal of sample and calibration volumes, washed in multiple refill operations. The procedure is then repeated for labelling and for the color reporting step, which leads to not less than 4 pipette tips and equal number of transfer pipettes for each well, plus the associated number of individual manual operation (Fig. 1a).

A field deployable version of such concept, or any protocol of equivalent complexity, should simplify the procedure, integrate volume metering, isolate all reagents from the environment, integrate the reagents within the device, accept modular functionalization, and eliminate auxiliary consumables.

In this work, we show such possibility through the simplification of the fluidics to the most elementary, and yet unconventional architecture, which allows the integration of active injection into disposables.

## Results and Discussion

Attempts at integrated immunoassays, such as ELISA, in LOC format have relied on demanding architectures that required specialized fabrication facilities, and modifications of the familiar protocol used with microplates^3,8,10^.

In contrast with those strategies, the current concept aims at respecting as close as possible the standard procedure of exposure-incubation-washing of the conventional ELISA, in order to facilitate a direct migration of the numerous ELISA chemistries commercially available^15^. Concurrently, the proposed architecture should eliminate those aspects that make the classical protocol too complicated for distributed scenarios, reduce reagent consumption, and limit the solution to a single disposable element without ancillary disposable elements. Such device should also produce a quantifiable response unaffected by casual illuminating conditions.

The devices in this work were fabricated as unibody-LOCs (ULOC)^16–18^, which entails designs configured as monolithic stereo lithography (SLA) 3D printouts, following particular fabrication rules. ULOC channels are always open on one side to allow easy removal of uncured resin, which allows the fabrication of arbitrarily long channels with consistent geometry. The devices are finished by sealing the open sides with adhesive tape, thus drastically simplifying assembly efforts. Typical short fabrication times permit multiple design iteration per day and at a cost of USD$ 0.55/device, while printers compatible with the ULOC fabrication cost less than USD$ 4000. ULOC capabilities have been extended to the fabrication of bespoke optics^19^, and demonstrated for quantitative glucose and glutamate sensing^5^.

More advanced protocols, like ELISA, demand rethinking the device architecture. Valves and pumps are normally complex and challenging to integrate in LOC devices. Even prior ULOC check-valves required elastic elements individually mounted and introduced ~0.5 μL dead volumes, which limited the number and uses of such components^18^. However, if valves and injectors could be produced with the same effort and cost as a regular channel, it would grant new possibilities to simplify the entire design, while extending the ULOC capabilities to execute complex protocols.

Figure 1a schematically illustrates the proposed architecture. A unidirectional manifold connected to as many injector modules as necessary could deliver transport, sample and reagents volume metering, positioning, and confinement. The manifold design demands that none of the injectors malfunction, which requires a reliable design able to accommodate the limitations of the 3D printing process. The check-valves must also contribute a null dead volume to prevent contamination from and to the manifold, and assembly should be reduced to one single simultaneous operation for all valves, thus eliminating ancillary elements^18,19^, and concurrently minimizing the valves footprint. In these conditions, the otherwise arduous efforts and  skills necessary for classical fabrication^20^ would be transferred to the 3D printer and reduced to a simple assembly step, even allowing to deploy fabrication if necessary.

The ULOC implementation of the unidirectional manifold (Fig. 1c,d) employs embedded 3D printed connectors, for a tight fitting to plug-in silicone tubing, to create the finger pumping element and reservoir at the same time. Each injector module is completed with its individual check-valve connecting to the 1 × 1 mm^2^ cross section manifold. The valve body is made by a robust geometry of 1 mm diameter hole across the unibody bulk and a 500 μm long barrier to the channel. Figure 1c,d, indicate the printing direction for this device, which is relevant for ULOC design, since such architectures do not use auxiliary supporting elements or bases, in order to fully control the geometry and to reduce materials consumption. Thus, for the designed printing direction the manifold channel has a curved bevel to support a reliable buildup of the channel wall before the valves.

Each particular detection target can be defined by the functionalization of the detection chamber (Fig. 1c). In order to keep the design generic, and ready to be repurposed to different targets, the capture antibodies are immobilized in separated 3D printed elements (Fig. 1c), which are plugged into the ULOC detection chamber during assembly. The device is completed with a waste reservoir (*w*, Fig. 1d), able to collect the total volume of reagents used in the assay. A filter paper coil and an expanding channel design in the first waste chamber prevent any backward contamination, without introducing significant forward flow resistance.

The assembly procedure, which can be completed in less than 1 min, consists of applying the tape to ULOC’s top and bottom surfaces, plugging-in the functionalized element in the detection chamber, plugging-in silicone tubing elements, and loading the reagents, which were simply injected from 10 mL stock solution syringes.

For ELISA tests, sample (*s* in Fig. 1d) is injected into the manifold and an aliquot is measured as the distance between injectors (5  μL). Once the sample volume is measured, it is positioned in the detection chamber using the air injector (*air* in Fig. 1d) and left in position to incubate. If a mistake is made during such process, the assay can be saved by aborting the incubation and transferring the aliquot to the waste reservoir, before repeating the operation. The current design also incorporates a simplified volume metering alternative, since reagents can be directly pumped into the detection chamber and then the excess volume displaced with the air injector. Thus, the detection chamber measures the volume, by isolating the tail connecting to the waste reservoir with a geometric barrier (Fig. 1d).

After the incubation time, a measured volume of washing solution (*w*_1_ *or w*_2_) is injected and flushed through the manifold using the air injector. Subsequently, the whole volume metering-incubation procedure can be repeated for the enzyme labeled antibody (*l*) and finally to incorporate the substrate for the label enzyme, which produces the quantitative color response.

Quantitative color detection can be easily contaminated by ambient light, and requires shielding the devices during measurements and providing a well-defined light source^19,21,22^. In this work a 3D printer sample holder secured the position of the device for video acquisition with the rear-side camera of a cell phone, while shielding from ambient illumination. Continuous flash illumination and controlled acquisition settings are available via free applications such as the OpenCamera app used in this work.

Essential to the device operation, is the mechanism of the check-valves detailed in Fig. 1e. To operate the injector the solution loaded silicone tubing is pressed, thus driving the flow in the forward direction. Without the presence of the check-valve the flow would reverse when the tubing is released, but the valve prevents backward flow, creating an effective forward flow every time the tubing is pressed. When the tubing is pressed, it generates enough pressure to lift the tape at the 500 μm long barrier to the manifold, whereas after the pulse has passed the elasticity of the tape reseals the barrier preventing the reflow, and creating an asymmetric pressure-flow configuration.

If one single valve fails, it provides a low resistance path that disables the whole device. The current system enables testing the backward sealing during assembly by pressurizing the air injector before the two vents in the second waste chamber are punched (Fig. 1c).

A central element to enable the injectors mechanism is the passivation of the tape glue over the gap. The glue cannot simply be removed because it contributes its elasticity to the sealing of the connection while the valve is idle. The solution presented here consist on a laser cut non-adhesive film mask, which is applied to the tape glued side. A simple squeegeeing of silicon grease thought the mask, enables a systematic and reliable procedure to passivate the tape with a 100 μm thick grease film (Fig. SI1). After removing the mask, the tape is simply aligned to the  ULOC injectors and adhered to its surface.

Alternatively, to this procedure, local coating with mineral oil of the gap region is also possible, but is less systematic and requires more practice to produce reliable results.

**Figure 2:**
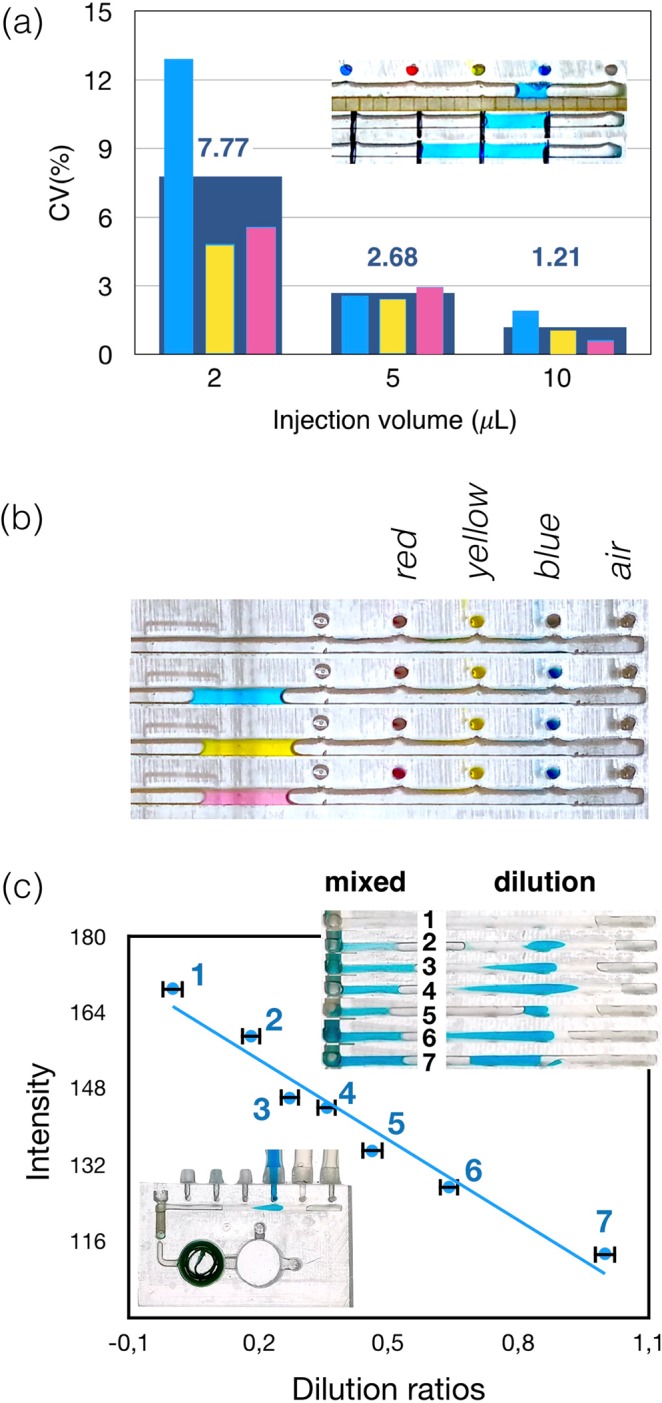
(**a**) Reliability of the injectors expressed as coefficient of variation for injection volumes between 2 and 10 μL. (**b**) Zoomed area of the manifold underscoring the null dead volume check valves characteristic as the absence of contamination between injectors and manifold when 5 μL volumes flow along the manifold. (**c**) Performance of the unidirectional manifold for sample dilution.

Volume metering is a critical operation for any analytical procedure, and in the classical ELISA format is performed using pipettes and numerous disposable pipette tips. The pipetting performance for the 1–10 μL range can be characterized be a coefficient of variation (CV), which is between 10 and 1% respectively^23^. Figure 2a shows the performance of the ULOC injectors delivering 2, 5 and 10 μL aliquots respectively. The figure collects the performance of injectors, which was corroborated by all experiments in this work (Fig. SI2 and videos in SI). The CV values closely follow those of commercial pipettes^23^, whereas in this case the injectors are integrated in the ULOC, do not need complementary disposables, and once the devices are loaded it can be operated in a dirty environment. The response that maters in these devices is that provided by the finger action; however, to establish the operational limit the injectors were tested with 50 ms pulses of 1Psi, for which they are capable of delivering 0.29 μL (Fig. SI3).

Null dead volume valves are indispensable to prevent contamination from and to the manifold. The achieved null volume characteristic can be observed in all Supplementary Videos, and is summarized in Fig. 2b, which zooms into one example of sequential injection of blue, yellow and red solutions. The transported volumes (air injector) in the manifold do not affect the color of the injector lines or become stained when passing the injectors locations, which illustrates the null volume exchange.

3D printed fluidics allows flexible aspect ratios, without the depth limitations in classical LOCs fabrication, often limited by planar characteristics of photolithographically generated templates. ULOC configurations in contrast can operate dynamic injection profiles with average flow rates in the order of 5 μL/s (Fig. SI4), and avoid mixing limitations^24,25^. In the current design, the dimensions and operation regime facilitate mixing within the manifold (Fig. SI5 and Video_11), which enables the possibility to perform competitive ELISA in the same platform.

Effective mixing allows to convert the unidirectional manifold into compact platform for sample dilution. Figure 2c illustrates a range of dilutions produced by sequential injection of different sample/solvent volume ratios in the manifold (Video_12 in SI). All dilutions are evaluated at the end of the manifold by its color intensity. The error bars correspond to the CV for 5 μL and the dilution relations are computed by the count of blue pixels in 1700-pixel regions.

**Figure 3:**
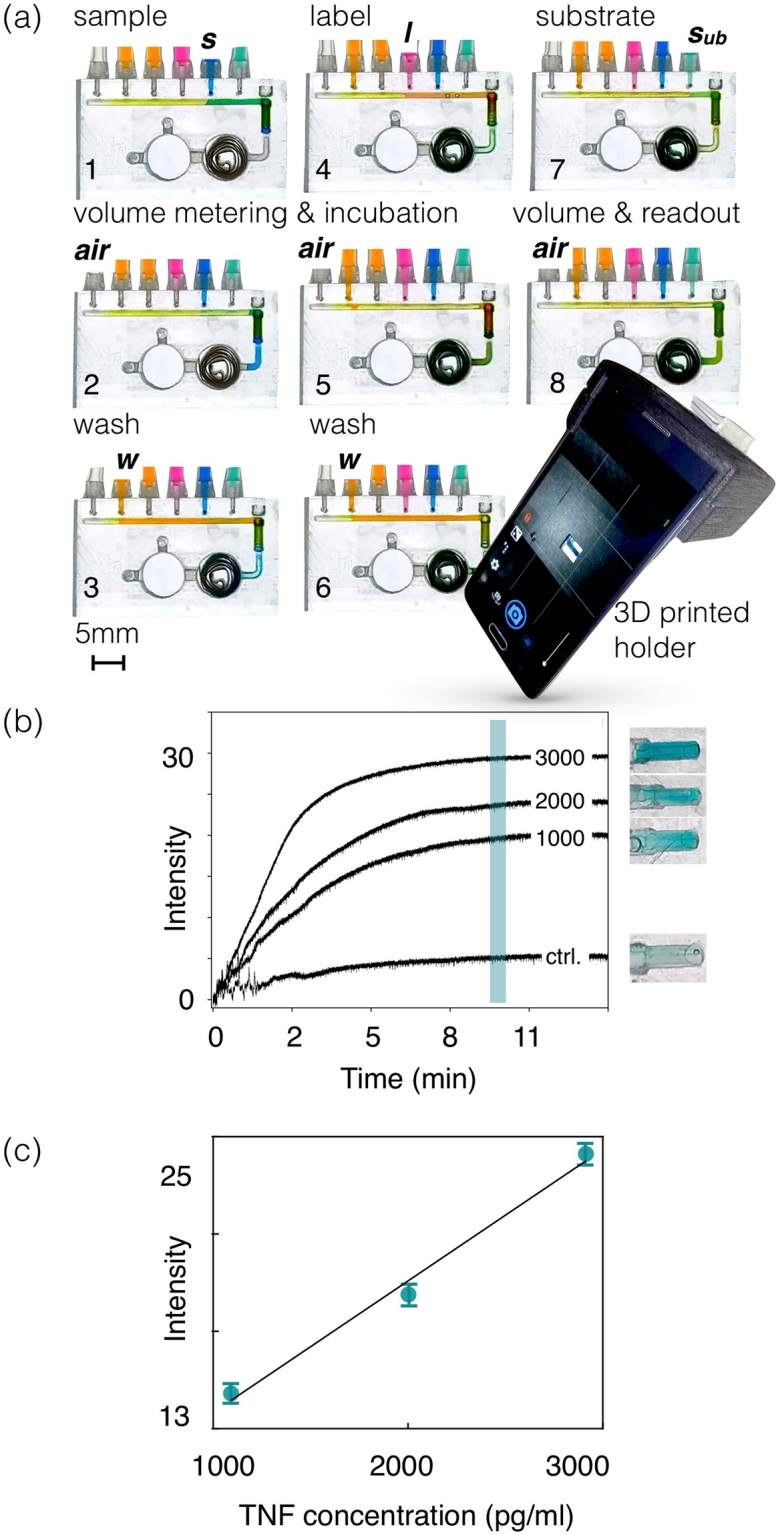
(**a**) Sequence of operations for a sandwich ELISA protocol as implemented in the proposed device. (**b**) Dynamic response of a commercial TNF ELISA assay video recorded at 30 fps. (**c**) Quantification of commercial TNF-ELISA for different analyte concentrations. Error bars correspond to 95% confidence interval.

As summarized in the introduction a standard ELISA protocol involves multiple operations, which must be implemented in the ULOC. Figure 3a collects these steps (see Video_13 in SI). In an assembled ULOC device, with all reagents integrated, the user only requires to introduce the sample and manual finger pumping, which replace all pipetting and washing procedures associated with a regular ELISA test. In this case, such simplification is not associated with a specialized protocol, but with the rather direct migration of commercial ELISA kits into the disposable device.

Accordingly, sample (*s*) is injected in the manifold and positioned (*air* injector) on the detection zone (Fig. 3a1), where it incubates for a determined interval (Fig. 3a2,a3). Subsequently, washing solution (*w*_1_, Fig. 3a4) is flushed through the detection zone. Label (*l*, Fig. 3a4) is then injected and positioned at the detection zone (*l*, Fig. 3a5) for incubation. After further washing (*w*_1_, Fig. 3a6) the manifold is vented (air injector), and finally substrate (*s*_*ub*_) is injected leaving the device ready for readout (Fig. 3a7–8).

Functionalization is provided by a plug-in element containing the capture antibodies for a given target. In this modular way the same architecture can be repurposed to diverse ELISA targets by choosing the plug-in and the associated reagents.

The current device is thus an autonomous platform able to integrate all reagents and actuators in a single disposable unit prepared for the manual execution of a regular ELISA protocol.

The device in this work was tested with a modified commercial ELISA assay for human soluble Tumor Necrosis Factor (TNF) receptor 1^26^. TNF receptor 1 is a ubiquitous transmembrane receptor that binds tumor necrosis factor-alpha (TNFα), which is a key mediator in inflammatory response and is implicated in the onset of a number of diseases^27^. The soluble TNF-R1 decoy form of the receptor is released into circulation via shedding of membrane receptors and elevated levels are associated with disease activity in several infectious, inflammatory and autoimmune disorders like systemic lupus erythematosus^28^, paracoccidioidomycosis^29^, kidney disease^30^ and untreated colorectal cancer^31^.

The color response of the detection region is captured in video at 30 fps. A region of interest comprising the detection chamber (Fig. 3b) is used to monitor the intensity of the color response. To compute the response vs. TNF concentration 1000 frames of the time response were integrated, at the 10 min mark set as the endpoint of the readout. This information defines the amplitude of the response and the error bars corresponding to 95% confidence. The linear response of the tested range is consistent with the TNF response in microplates (Fig. SI6a,b). The only difference is the more effective geometry of the ULOC device that enables readout after 10 min, rather than the 35 min demanded by the microplate implementation.

## Conclusions

In a compact and modular configuration, the demonstrated device offers the ability to integrate and run bioassays with the complexity of ELISA protocols. Such capability is conferred by a robust architecture and fabrication procedure that navigate the virtues and limitations of 3D printed LOC devices and contributes a design that integrates a unidirectional manifold of 6 injectors into a disposable device for USD$ 0.55. Thus, effective mixing, volume metering, arbitrary dilution rates and complex bioassay protocols can be implemented in a same format supporting multiple and complementary uses. The simplicity of the assembly procedure also highlights the possibility of deployable fabrication capabilities.

Active injection combined with volume metering is one of the most humble and robust tools serving biomedical and analytical uses since Roman times^32^. The present concept re-interprets and expands the capabilities of the classical syringe to cover the demands of the current century.

## Methods

### ULOC fabrication

ULOC devices were designed with free computer aided design (CAD) software (Autodesk Inventor Fusion, Autodesk Inc.) and fabricated with a Form1 + 3D printer (Formlabs Inc., USD$ 3400), a 405 nm laser source stereo lithography (SLA) platform.

Unibody LOC (ULOC) assembly procedure has been described elsewhere^5,18^, and consist of designing a monolithic geometry containing all the necessary features to host functionalization and actuation. All geometrical features are designed with one open side to ease the removal of uncured resin and to allow arbitrarily long channels.

After printing, the devices were sonicated (FinnSonic m15, FinnSonic Oy) in industrial-grade ethanol for 20 s and air-jet dried. To seal the open sides of such ULOCs adhesive tape (3 M Ruban Adhesive Scotch Nastro Adhesive, 3 M Europe, Diegem, Belgium) was used. It is worth noticing that adhesive tape is a convenient choice, but ULOC can be sealed with other types of films and glass.

Before attaching the tape to the ULOC the surfaces were polished with sand paper (Silicon Carbide P#500, Struers Sverige)

The printer uses a proprietary resin (Formalbs Clear Type02, US$ 163/L), which formulation is not disclosed but entails a modified acrylate oligomer and monomer in combination with an epoxy monomer, a photo initiator and additives^33^. For this resin cost, the 3.41 mL devices in this work corresponded to US$ 0.55/device.

ULOC offers excellent development costs and grants the versatility of producing several iterations a day of any particular design. However, once the architecture is optimized the natural step is to transfer to a mass manufacturing method that secures lower costs per unit and the selection of materials that guarantee long-term stability in contact with the reagents.

The assembly procedure consists of applying the tape to both sides of the ULOC, plugging-in the functionalized detection region using a tweezer, and sealing it with the printer resin, which in this case is cured *in situ* a 100 mW 405 nm laser.

In order to passivate the tape in the region that covers the gap between the injectors and the manifold, tape patterned with silicon grease was used. A laser cut mask (HL40-5g, Full Spectrum Laser LLC) in non-adhesive material was attached to the glued side of the sealing tape. Through the openings in this mask, which were defined by the pitch and geometry of the injectors’ layout, silicon grease was squeegeed. This procedure, once the mask was removed, defined a repeatable pattern of 100 μm thick grease patches, which were then aligned with the valve orifices when the tape was assembled to the unibody (Fig. SI1).

Although, the procedure requires a mask, such element is reusable and delivers very repeatable results that demand less fabrication skills, than local coating of the gaps with mineral oil, which is another possibility.

The device was finished by plugging-in the silicon tubing in the unibody connectors, after which the reagents were loaded using syringes with stock solutions.

### Injectors characterization

Quantitative data in this work were obtained from the processing of video acquisitions. Videos were acquired using a Galaxy Note 2 (Android KitKat) rear side camera operating at 1920 × 1080 pixels resolution and 30 fps, and a Motorola Moto G5S (Android 8.1.0) operating at 1280 × 720 pixels resolution and 30 fps. The video acquisitions were controlled with the free application OpenCamera v1.44.1.

The quantification of injection volumes was performed from video acquisitions. Selected frames were captured as.png images and analyzed using Image J 1.50i.

The quantification of injected volume variability consisted of repeated injections of 2, 5, and 10 μL volumes into the manifold using the integrated finger pumps. Evaluations were made using all injectors in a same device for each volume range, in order to test the simultaneous operation of the injectors, which is critical to establish the robustness of the design.

Fig. SI2 illustrates the measuring procedure for 5 μL injections. In this case 4 consecutive injections were registered for the blue solution (Video_01). After each injection, the 5 μL volumes (5 mm distance between injectors and 1 × 1 mm^2^ manifold cross section) were displaced using the air injector. In order to properly quantify the volumes, video frames showing the image of such volumes were collected (Fig. SI2).

The same procedure was repeated for the yellow solution (Video_02), and red solution (Video_03) using the same device. Measurements of 2 μL and 10 μL volumes (Video_04 to 09) followed the same procedure. The quantification of such volumes is collected in Fig. 2a and the characterized behavior was consistent across all devices tested in this work.

Collected images were split in color channels and the best contrast for each color substance was thresholded in Image J to produce black and white  images. From these binary images a fixed area was used to compute the histogram representing the volume in each image. The number of black pixels was thus used to assess the variability of each delivered volume.

The coefficient of variability (*CV*) for each injection data set (*X*) was computed according to:

$$CV( \% )=\frac{std(X)}{mean(X)}\ast 100$$

CVs notoriously over-represent sets with small mean values, but is a simple measure to compare performance with commercial pipettes^22^.

As shown by the current analysis (Fig. 2a), the ULOC injectors closely follow the performance of regular pipettes, but with a simpler operation, avoiding tip exchange, and saving the additional tip consumption.

Despite of that the relevant aspect of the injector is to operate under finger pressure, and not the actual value of such pressure, we characterized the injectors at a known pressure value in order to establish the operational limit, which can be used to compare devices produced by this method.

In this case, the pressure source was provided by a pressure micro-injector (PMI-200, Dagan Corporation). Pressure pulses of 1 Psi. and 50 ms of duration were applied to the same injector, while the response was recorded in video (Video_10). Video frames showing the stabilized volume present after each pulse were collected and assembled in Fig. SI3. After thresholding, black pixel counts defined an average injection volume of 0.29 μL/pulse.

Regarding the air injection, which is the transport and positioning mechanism used in this device, the maximum achievable flow rate can be estimated by two actuations of the air injector on a 5 μL volume, showing an average peak flow of 5.75 μL/s (Fig. SI4).

It is worth noticing that such average flow (averaged over 2 s interval) is smaller than the maximum flow at the injection instant, when the elasticity of the tape seal must be overcomed to allow the connection to the manifold. Even in this condition of maximum pressurization of the manifold, there is no connection from and to the inactive channels, due to the null dead volume design that secures uncontaminated operation (Fig. 2b, all Videos).

The dynamic of such injection and the dimensions and aspect ratios achievable with ULOC, and other 3D printed systems^18^, have the collateral advantage of simplifying mixing. Mixing in classical microfluidics is demanding due to the predominant laminar flow regime in microchannels operated at constant flow rates^24,25^. Practical limitations in the depth of microfluidic channels, given by the planar photolithographic techniques and materials used for templates, originate these limitations and define the tolerable pressures and achievable flow rates. In contrast, ULOC fluidic channels can be several millimeters deep, and as in this case operate in a transient regime, which facilitates mixing.

Figure SI5 (and Video_11) illustrates such effective mixing behavior in the considered device. Successive injections of blue (10 μL), yellow (5 μL, for contrast to highlight the yellow injection profile), red (5 μL) and blue (5 μL) volumes is collected in Fig. SI5a. Air transport at 7 s, and 11 s intervals, show mixture after this period, without additional mixing architectures required in the design.

Another important application of effective mixing is to create arbitrary sample dilution ratios integrated in the device. Different mixing ratios were quantified by thresholding selected frames from video acquisitions, and counting the proportion of blue pixels in the total of each mixture. The resulting dilution was characterized by the blue intensity of the mixture at the end of the manifold (Fig. 2c).

### Human sTNF-R1 assay

Human TNF RI/TNFRSF1A DuoSet ELISA kit was purchased from R&D Systems, Inc (DY225) and the assay was performed in 96 well microplates according to the manufacturers protocol, with two modifications: (1) The detection antibody was a polyclonal goat human TNF R1 antibody (R&D Systems, Minneapolis) to which horse radish peroxidase (HRP) was conjugated using the Lightning-Link HRP conjugation kit (Novus Biologicals, LLC) according to protocol (used working concentration was 0,4 µg/ml) and (2) tetramethylbenzidine substrate solution (Amresco # J644) was used without stop solution.

For the ELISA within ULOCs, plugins were functionalized with 50 µl human TNF R1 capture antibody (R&D Systems, Inc) at 4 µg/ml in PBS pH 7,4 overnight, washed in wash buffer (0,05% Tween in PBS pH 7,4) and then blocked for 1 h in 1% BSA in PBS pH 7,4 (reaction buffer) to prevent unspecific binding.

To establish the operating conditions and calibration range simplified ULOCs were used (with identical chamber and plug-in element) and plugins were incubated with different analyte concentrations (recombinant human TNF R1 standard, 0,5 ng/ml, 1 ng/ml, 2 ng/ml and 3 ng/ml in 50 µl reaction buffer, or 20 µl and chamber metering in the real device) for 1 h, washed by dipping 3 times (or 40 µl flow) in wash buffer and then incubated for 1 h in detection antibody (0,4 µg/ml, 50 µl, or 20 µl in the device and chamber metering). After a washing step (8 times dipping, or a 50 µl flow), the plug-ins were inserted in the simplified device, and substrate solution was pumped into the reaction zone (20 µl and chamber metering).

### Assay readout

The detection region of either the ELISA in a 96 wells microplate or in ULOC devices were video recorded under controlled illumination and fixed acquisition conditions (ISO 100, 1280 × 720 pixels, 30 fps, locked exposure, fixed focus) commanded by the free application OpenCamera.

Videos were processed with a bespoke Python 3.7 program, using the opencv 3.4.2 environment for video analysis. The code enables to align regions of interest (e.g. in Fig. SI6b) to capture the intensity of the red camera channels to follow the time response of the TNF assay.

Enpoint responses in the calibrated ranges were composed by averaging 1000 frames at the 10 min mark for the case of ULOC based detections and at 35 min for the microplate-based tests.

Error bars were computed as the standard deviation in these conditions and represented for 95% confidence.

## Supplementary information

Supplementary Information

Video 01

Video 02

Video 03

Video 04

Video 05

Video 06

Video 07

Video 08

Video 09

Video 10

Video 11

Video 12

Video 13
