# Supplementary material for: Autonomous lab-on-a-chip generic architecture for disposables with integrated actuation
Source: Sci Rep. 2019 Dec 30;9:20320. doi: 10.1038/s41598-019-55111-z (PMC6937297; doi:10.1038/s41598-019-55111-z)
Supplement: Supplementary file 1 — Supplementary Information [file 41598_2019_55111_MOESM1_ESM.docx]

# Supplementary Information:

# Autonomous lab-on-a-chip generic architecture for disposables with integrated actuation

Anke Suska, Daniel Filippini*

Optical Devices Laboratory, Division of Sensor and Actuator Systems, IFM-Linköping University, S58183, Linköping, Sweden.

*e-mail: [daniel.filippini@liu.se](mailto:daniel.filippini@liu.se)

## Contents

Video_01: 5 μL injections, blue solution.

Video_02: 5 μL injections, yellow solution.

Video_03: 5 μL injections, red solution.

Video_04: 2 μL injections, blue solution.

Video_05: 2 μL injections, yellow solution.

Video_06: 5 μL injections, red solution.

Video_07: 10 μL injections, blue solution.

Video_08: 10 μL injections, yellow solution.

Video_09: 10 μL injections, red solution.

Video_10: Successive injections of blue solution at 1PSI.

Video_11: Mixing efficiency.

Video_12: Dilution experiment.

Video_13: ELISA protocol.


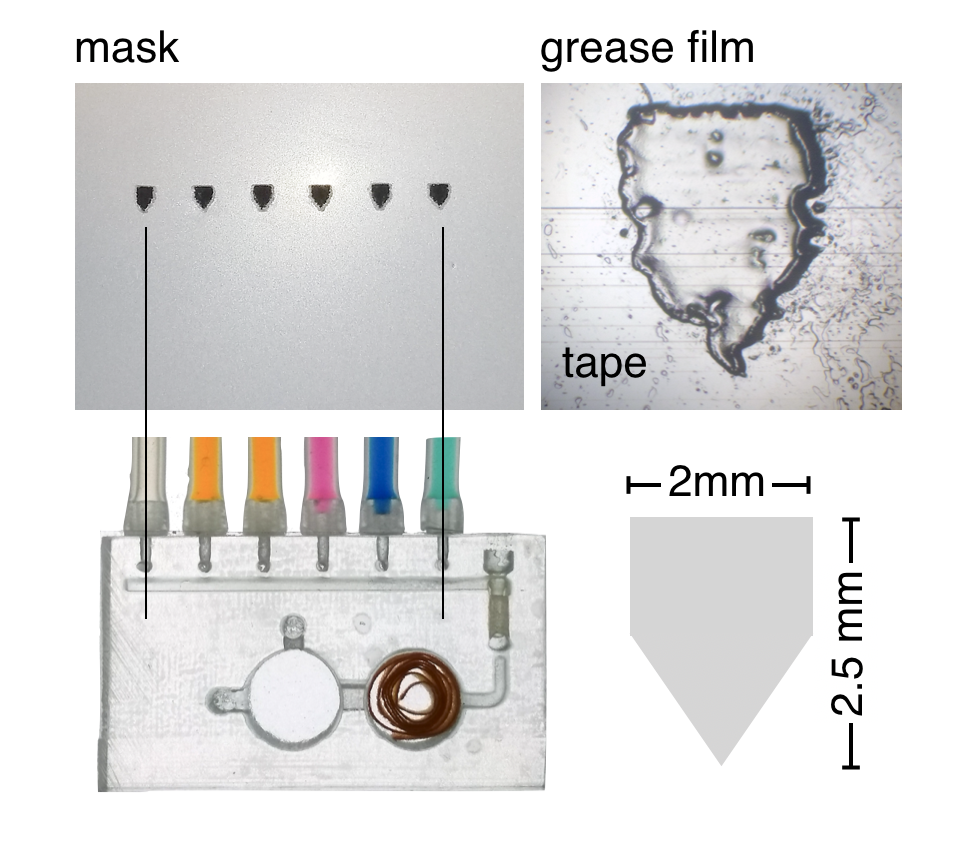


Fig. SI1. Laser cut mask on non-adhesive film used to squeegee silicon grease to the sealing tape adhesive side. The mask layout matches the position of the injectors orifices and passivate the gap region necessary for the check-valve operation.


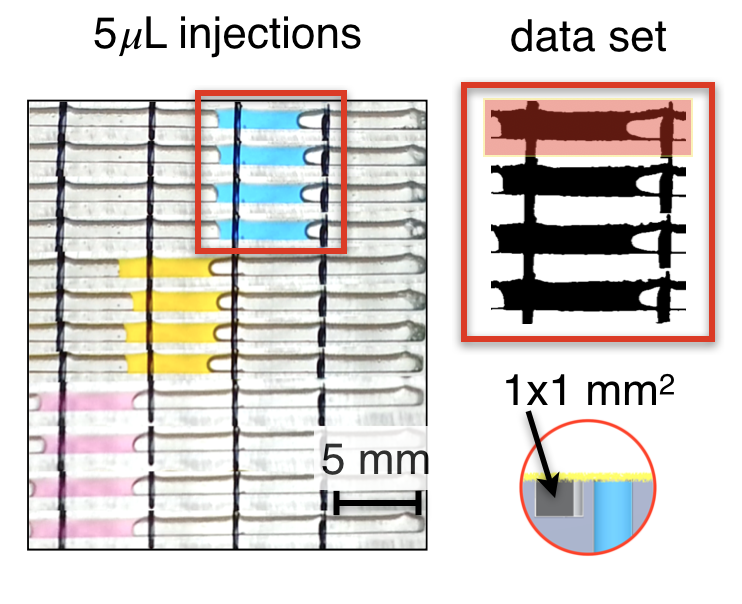


Fig. SI2. Collection of repeated 5μL injections of the blue, yellow and red solutions on a same device, and the resulting binary image of the blue data used to compute the volume of each injection. The manifold cross section is 1 x 1 mm^2^. (Video_01-03).


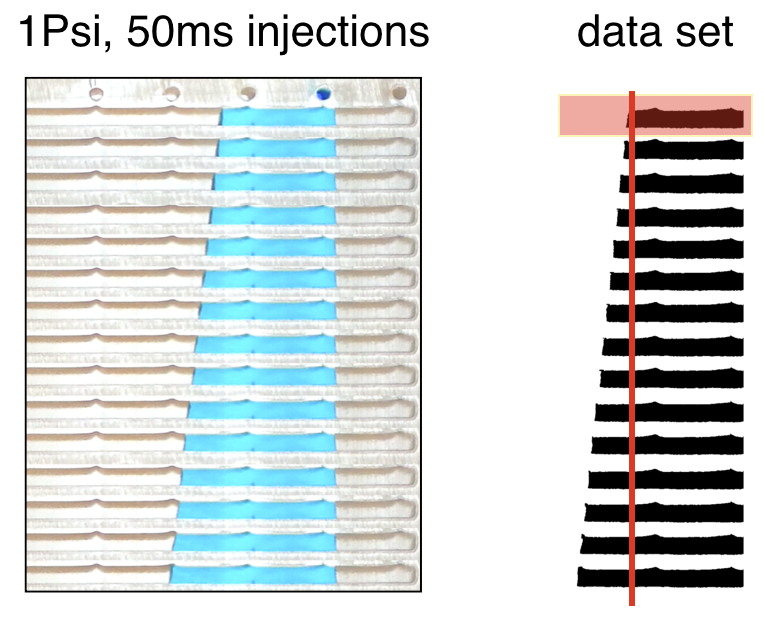


Fig. SI3. Sequential injections using 1Psi pulses of 50ms and the binary image used to the determine the average volume injected in these conditions (Video_10).


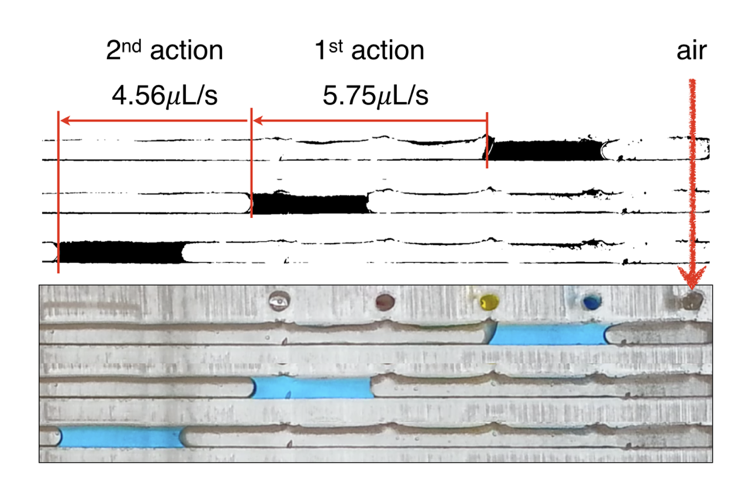


Fig. SI4. Maximum flow rates due to finger pumping of the air line against a 5μL volume after a first and second actuation.


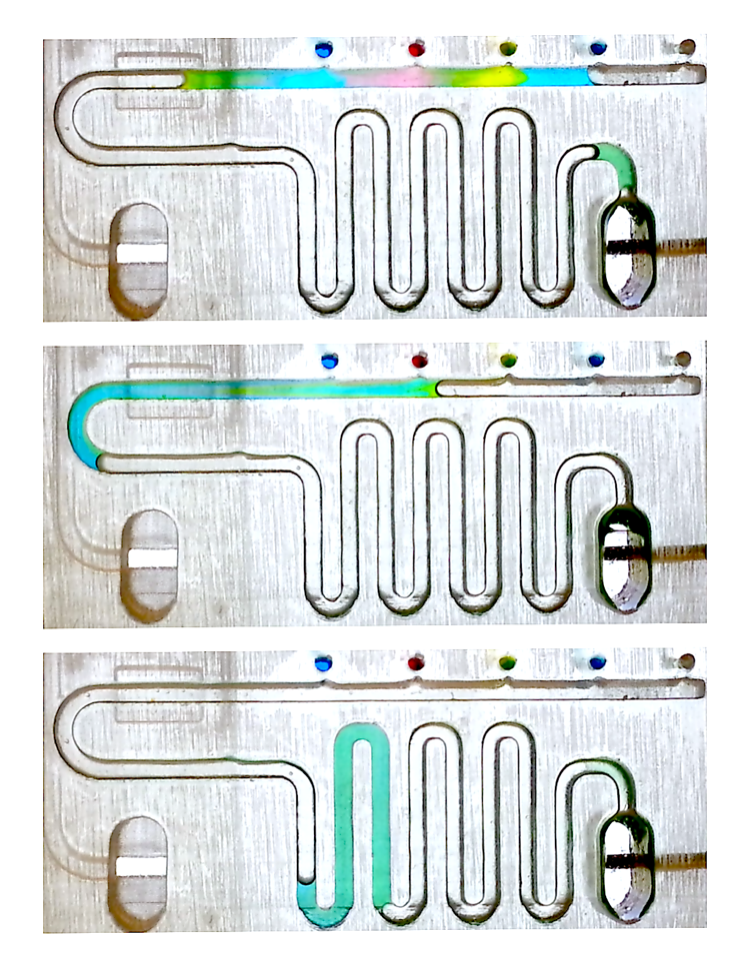


Fig. SI5. Mixing performance after injection of 4 color solutions in the manifold and transport using the air injector. (Video_11).


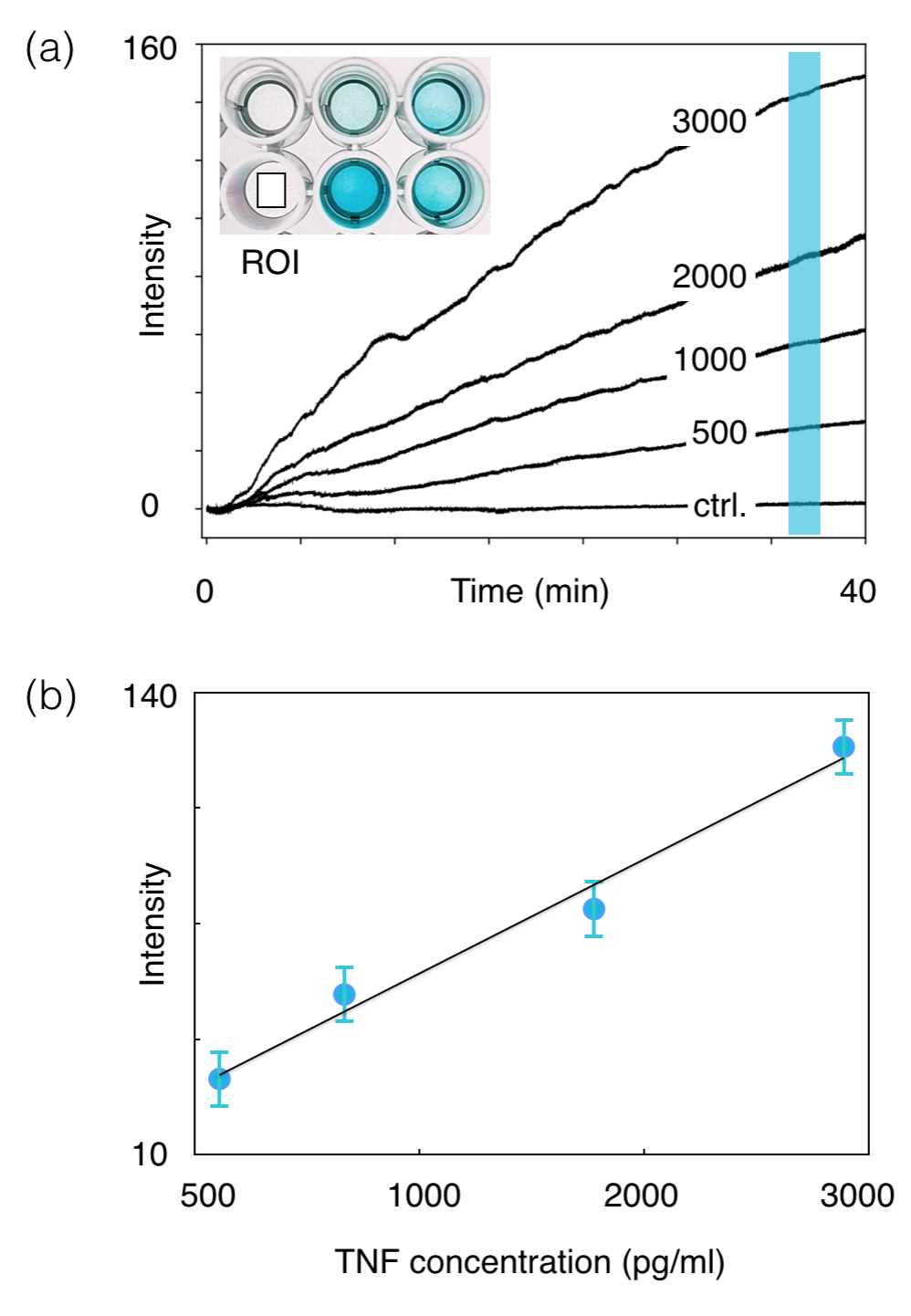


Fig. SI6a) Dynamic response of a commercial TNF ELISA assay video recorded at 30 fps. b) Quantification of commercial TNF-ELISA for different analyte concentrations measured on a 96 wells microplate (Insert). Error bars correspond to 95% confidence interval.
